# Items of General Interest

**Published:** 1918-09

**Authors:** 


### NEWS ITEMS.

A hospital unit designed exclusively for work among gas victims, the first of its kind and recruited from many cities for the Women's Overseas Hospitals, the hospitals of the National American Woman Suffrage Association, has started for France, it is announced.

Dr. M. E. Taber, Medical Director of North Dallas Exemption Board, in a statement recently issued, said that the industries will be considerably handicapped after the Government inducts many active medical men into military service. Some plan of centralization of medical supervision, as well as handling surgical cases, might become necessary. Many of the exemption board are losing their medical members. He favors older men on the boards or some arrangement whereby the experience of those who have served a year or more on these boards should not be dissipated.

The United States will pay $300,000 a year for a store building in New York City, in which is to be established a hospital for medical cases among soldiers brought back from France. Accommodations for 50,000 soldiers are being sought in the city by the government. Most of these will be used for the sick and wounded, but quarters will also be necessary for the attendant surgeons, nurses and helpers.

The Government will in the future make arrangements for free surgical operations for those men whose induction into the army has been deferred because of remedial defects, providing they are willing to submit to such operations in order to join the colors, according to letters received by the local exemption board from Major John C. Townes, Jr., supervisor of selective service in Texas.

Questionnaires are now being mailed out to all regularly licensed practicing physicians which will be used as a basis to determine the eligibility of the physicians for service in the medical reserve corps. All physicians accepted for service will be commissioned with a rank of not lower than a first lieutenant. The questionnaires have been furnished by the medical council of national defense at Washington. The questionnaires of the physicians do not come under the jurisdiction of the district exemption board, but are to be handled by the State Medical Council of Defense, whose chairman is Dr. Ira Carleton Chase of Fort Worth, to whom all questionnaries should be sent. These questionnaires will be finally passed upon and the physicians selected for service by the governing body for Texas of the Volunteer Medical Service Corps, consisting of Dr. Bacon Saunders of Fort Worth, Dr. R. W. Knox of Houston, Dr. J. E. Gilcreest of Gainesville, Dr. W. L. Brown of El Paso and Dr. Frank Paschal of San Antonio.

More than 18,000 Fort Worth citizens have taken advantage of the anti-typhoid serum since the establishment of a free clinic, and there seems to be no doubt that the bureau will inoculate the desired 35,000 before the close of the season. Dr. J. G. Townsend has been informed from Washington that seven quarts of vaccine will be sent to Fort Worth each week for use by the clinic. At the usual rate of 1,000 inoculations per quart, 7,000 people can be given the treatment each week.

Dallas physicians are firmly establishing an unrivalled record for patriotism and unselfish public service. Perhaps a larger percentage of local doctors have volunteered for hospitl service overseas than has occurred elsewhere in the Southwest, and recently it was disclosed that local physicians have in all instances been refusing to accept fees for the examination of hundreds of applicants for training camps and other branches of service. In some cases practically all of a physician's time is taken up in such unrenumerative work, it has been said. In addition to the above several local specialists recently volunteered for service to the United Charities for difficult cases that may come under the care of that association.

The cause of Empegma, the disease of the lungs which assumed the proportions of an epidemic in the training camps and cantonments last winter with a large number of deaths has been ascertained and the best methods of treatment established Surgeon General Gorgas announced today. The new treatment consists of removing the fluid formed in the lungs by aspiration.

After six months' trial of the women's overseas hospitals, the French government has asked the National Woman Suffrage association, which sent the unit over and is financing it, to supply immediately a personnel of 50 women, doctors, nurses, nurses' aids, clerks, chauffeurs, etc., to run a 300-bed hospital to be established for the care of gas cases. Mrs. Raymond Brown of New York, who went to France for the suffragists to inspect the units they had sent and report what was needed, came back with this official request and is scouring the country now for the very best experts her sex can furnish to enlist for this dangerous work.

Dr. H. C. Hall of Laredo has been appointed by the surgeon general of the United States to the position of director of the veneral disease control for the State of Texas.

Dr. Hall was recommended by State Health Officer W. B. Collins and had the endorsement of Governor Hobby.

Dr. Hall and Vice Consul Randolph Robertson, representing the United States in the City of Mexico, have gone to Washington, D. C., to discuss the Mexican labor problem.

